# Is the Human Eye Changing Its Form?

**Published:** 1878-02

**Authors:** 


### Is the Human Eye changing its Form, and becoming Near sighted, under the Influence of Modern Education?

Dr. E. G. Loring recently read an interesting paper on this subject before the New York County Medical Society (*New York Med. Journ.*, Dec. 1877). He said that hereditary influence was an important element in the production of myopia, and, although statistics did not strongly indorse that view, he still held that legendary information should receive much credence. In regard to the influence of modern education, it was found that a larger proportion of those living in cities were near-sighted than those in country districts; and, moreover, in those cities where intellectual pursuits were greatest, the largest number of myopes were found. In savage nations near-sightedness was very infrequent, and it would seem, in some respects, that it was a result of education. While the intellectual classes in Germany showed a large proportion of myopia, it was not so found in those artisans who used their eyes on fine objects, as watch-makers and wood-engravers. In England, where there has always been great intellectual activity, by no means as large a ratio of near-sightedness had been detected as in Germany, and it became necessary to seek for other factors to explain the prevalence of myopia. Impaired nourishment, imperfect ventilation, together with a sedentary life, had a marked tendency in producing laxity of the tissues in general, including of necessity the coats of the eyeball; and, with the tension which resulted from close application of the sight, there was a great probability of lengthening of the eye, or myopia, resulting. In New York the German children were found more often near-sighted than those of other nationalities. Dr. Loring said that undoubtedly myopia was hereditary, but that in all probability it could under no circumstances be developed; but he did not believe that of necessity it must increase in a nation engaged in literary pursuits. In the United States the normal eye predominated, and he thought it was due to the fact that the young were more in the habit of indulging in out-door sports than in Germany. The same was true of England. From a careful analysis of the myopic cases, it was found that between the ages of ten and fifteen the majority developed; or, in other words, at that time the tissues of the globe were more readily affected by strain of the muscles of the eye. It could be easily understood, under such an hypothesis, why the industrial classes were so little liable to near-sightedness, for they seldom reached the practice of the more intricate branches of their trade before their eighteenth year. In conclusion, Dr. Loring was of the opinion that, under proper precautions, the normal eye could be continued indefinitely. If children were not allowed to apply themselves too closely to their studies between their eighth and sixteenth years, and were, moreover, allowed the proper amount of out-door exercise, not much danger need be dreaded. It was important also to have the schools perfectly ventilated, and other hygienic conditions made as perfect as possible.—*Medical News and Library.*

